# Application of Novel Plasmonic Nanomaterials on SERS

**DOI:** 10.3390/nano10112308

**Published:** 2020-11-22

**Authors:** Grégory Barbillon

**Affiliations:** EPF-Ecole d’Ingénieurs, 3 bis rue Lakanal, 92330 Sceaux, France; gregory.barbillon@epf.fr

During these past two decades, the fabrication of ultrasensitive surface-enhanced Raman scattering (SERS) substrates has explosed by using novel plasmonic materials such bimetallic materials (e.g., Au/Ag) [1,2,3,4], hybrid materials (e.g., metal/semiconductor) [5,6,7,8], and also new designs of plasmonic nanostructures (e.g., nanoparticle self-assembly [9,10,11]). These novel plasmonic nanomaterials can allow a better confinement of the electric field and thus induce an enhancement of the SERS signal (electromagnetic contribution [12,13]) by adjusting, for instance, the size, shape, periodicity, nanoparticle self-assembly, and nanomaterials’ nature. These nanomaterials can also enhance the charge transfer (electrons; chemical contribution) to increase the SERS signal [14,15]. Furthermore, other materials are appeared for SERS applications such as metal oxides [16,17]. Other directions for the SERS field also emerged such as the SERS effect induced by high pressure [18,19], and the photo-induced enhanced Raman spectroscopy [20,21,22]. Thus, this special issue is dedicated to introducing recent advances and insights in these novel plasmonic nanomaterials applied to the fabrication of highly sensitive SERS substrates for chemical and biological sensing.

This special issue is formed of 5 research articles, and 1 review article. The first part of this latter is devoted to the novel methods of fabrication of plasmonic nanoparticles or nanostructures for SERS [23,24,25]. Dizajghrobani-Aghdam et al. demonstrated an alternative method of fabrication of metallic nanoparticles by employing pulsed laser ablation, and these hybrid plasmonic nanostructures have presented significant enhancements of the Raman signal [23]. Furthermore, Yang et al. presented the direct fabrication of SERS substrates by using an *in situ* photochemical method of reduction. High enhancements of the Raman signal were obtained with these SERS substrates [24]. To finish this first part, Chang et al. proposed a simpler method of electron beam lithography in order to realize SERS substrates by removing the photoresist lift-off step [25]. In the second part, the presented domain is devoted to the impact of long-range interactions and the surrounding medium on the SERS effect investigated by Ragheb et al. [26]. In the last part, the addressed domains are dedicated to novel plasmonic and non-plasmonic nanomaterials for SERS sensing [27,28]. Barbillon et al. demonstrated the enhancement of the Raman signal with hybrid nanostructures on a metallic film [27]. To finish this part and this special issue on novel plasmonic nanomaterials applied to the SERS field, Barbillon presented a short review on plasmonic and non-plasmonic nanomaterials for SERS sensing [28].

For performing the special issue entitled “Application of Novel Plasmonic Nanomaterials on SERS”, a couple of contributions has been obtained from excellent-quality authors originate from worldwide. I would like to acknowledge all these authors as well as the whole editorial office of the journal “Nanomaterials” for their great support and help in the management process of the article submissions and other associated tasks. To finish, I hope that you will find interesting this special issue devoted to novel plasmonic nanomaterials for the SERS field, which is targeted to the students or researchers who are or wish to imply in this field.
